# Physicochemical Characterization and Oral Bioavailability of Curcumin–Phospholipid Complex Nanosuspensions Prepared Based on Microfluidic System

**DOI:** 10.3390/pharmaceutics17030395

**Published:** 2025-03-20

**Authors:** Bo Zhang, Wenjing Guo, Zhenyu Chen, Yaxin Chen, Ruining Zhang, Minchen Liu, Jian Yang, Jiquan Zhang

**Affiliations:** Engineering Research Center of Modern Preparation Technology of TCM of Ministry of Education, Shanghai University of Traditional Chinese Medicine, Shanghai 201203, China; zb15670581612@163.com (B.Z.); wjguo2021@163.com (W.G.); chenzhenyu0829@163.com (Z.C.); shuiizhu@126.com (Y.C.); 13331798862@163.com (R.Z.); liuminchen@shutcm.edu.cn (M.L.)

**Keywords:** microfluidics, curcumin, nanosuspensions, nanomedicine, phospholipid complex, bioavailability

## Abstract

**Background**: Curcumin has been proved to have promising prospects in the fields of anti-inflammation, antibacterial, anti-oxidation, and neuroprotection. However, its poor water solubility and stability in strong acid, as well as fast metabolism, lead to low bioavailability, making it difficult to develop further. This study aimed to improve the bioavailability of curcumin by using microfluidic preparation technology. **Methods**: Using a self-built microfluidic system, polyvinylpyrrolidone K30 and sodium dodecyl sulfate were used as stabilizers to further prepare curcumin–phospholipid complex nanoparticles (CPC-NPs) on the basis of curcumin–phospholipid complex (CPC). The CPC-NPs were characterized and evaluated by X-ray powder diffraction (XRD), differential scanning caborimetry (DSC), dynamic light scattering, and transmission electron microscopy (TEM). Blood samples were collected from rats after oral administration of curcumin, CPC, curcumin nanoparticles (CUR-NPs), and CPC-NPs, respectively. The pharmacokinetics were analyzed by enzymatic digestion and HPLC. **Results**: The optimized CPC-NPs had a particle size of 71.19 ± 1.37 nm, a PDI of 0.226 ± 0.047, and a zeta potential of −38.23 ± 0.89 mV, which showed a spherical structure under TEM and good stability within 5 days at 4 °C and 25 °C. It was successfully characterized by XRD combined with DSC, indicating the integrational state of curcumin–soy lecithin and conversion to an amorphous form. The results of the pharmacokinetic study showed that the C_max_ of curcumin, CUR-NPs, CPC, and CPC-NPs were 133.60 ± 28.10, 270.23 ± 125.42, 1894.43 ± 672.65, and 2163.87 ± 777.36 ng/mL, respectively; the AUC_0–t_ of curcumin, CUR-NPs, CPC, and CPC-NPs were 936.99 ± 201.83, 1155.46 ± 340.38, 5888.79 ± 1073.32, and 9494.28 ± 1863.64 ng/mL/h. **Conclusions**: CPC-NPs prepared by microfluidic technology had more controllable quality than that of traditional preparation and showed superior bioavailability compared with free drug, CPC, and CUR-NPs. Pharmacodynamic evaluation of anti-inflammatory, anti-oxidation, and neuroprotection needs to be confirmed in follow-up studies.

## 1. Introduction

Curcumin [1,7-bis(4-hydroxy-3-methoxyphenyl) 1,6-heptadiene-3,5-dione] is a polyphenolic compound originally extracted from the rhizome of *Curcuma longa* L., which has been used for more than 4000 years. Modern pharmacological studies have confirmed that curcumin has anti-inflammatory [1], anti-microbial [2], anti-oxidant [3], and neuroprotective effects [4] and has the potential to treat Alzheimer’s disease [5] and many other diseases [6,7]. Meanwhile, the superior safety profile gives curcumin an adequate dosage range [8]. Unfortunately, its poor water solubility and stability in strong acids, as well as fast metabolism, lead to low bioavailability, limiting its clinical application [9,10]. Lu L et al. [11] found that 75% of curcumin was excreted in the excrement when an oral dose of 1 g/kg was given to rats, and the content of curcumin in serum remained very low, even undetectable, at an oral dose of 2 g.

Currently, promising approaches that have been shown to improve bioavailability mainly focus on the development of novel delivery systems, including but not limited to nanosuspensions [12], solid lipid nanoparticles [13,14], emulsions [15], micelles [16], liposomes [17,18], polymer nanoparticles [19], and phospholipid complexes [20,21,22]. Compared with other widely used nano dosage forms such as SLN, micelles, liposomes, polymer nanoparticles, etc., phospholipid complexes, as the star dosage forms in recent years, have more significant advantages. Firstly, phospholipid complexes have better biocompatibility and safety. This is because the prescription of phospholipid complexes is often only API and phospholipids, without the need to add the rest of the metal particles or surfactants, and phospholipids are components of cell membranes, have excellent biocompatibility, and have a low risk of toxicity, so phospholipid complexes tend to have better safety. Secondly, the preparation process of phospholipid complexes is relatively simple, often requiring only the conventional solvent volatilization method, without the need for complex equipment and high-pressure conditions. Thirdly, phospholipid complexes have a relatively high drug-loading capacity, which is conducive to improving drug safety, economy, and compliance. The phospholipid complex consists of polar functional groups of the active ingredient linked to the C=O or P=O of phospholipids through hydrogen bonding (not a mechanical mixture of the two substances) [23], which can be easily prepared to enhance the bioavailability of curcumin [24]. The most commonly used method for the preparation of phospholipid complexes is solvent evaporation, which has the disadvantages of being time-consuming (usually taking 2–4 h [24,25]) and having poor inter-batch consistency. Meanwhile, the semisolid nature and mediocre oral stability of such a dosage form may also limit its further application [26]. To overcome these shortcomings, there is an urgent need for a novel process for the preparation of curcumin–phospholipid complexes (CPC) and their nano-formulations that ensures uniform quality of the preparation.

In recent years, the emergence of microfluidic technology has provided new options for the preparation of nano-carriers. Microfluidics is a science and technology for precise control of microscale fluids, featuring fluid manipulation in micro- and nanoscale space, and can provide precise, reproducible, and controlled mixing at nanoliter scales [27]. The greatest advantage of this technique over traditional methods is the ability to control the variation of nanoparticles from batch to batch within a limited range while ensuring mono-dispersion [28,29,30]. Microfluidic devices expedite fluid mixing, ensuring optimal homogeneity before nucleation, thereby yielding nanoparticles with superior mono-dispersity; conversely, traditional methods allow nucleation, growth, and aggregation of nanoparticles to occur simultaneously, leading to poly-dispersity of the nanoparticles [31,32]. Because the function of nanoparticles depends greatly on the properties of the nanoparticles, such as particle size, shape, and zeta potential [33,34], all these properties can be regulated by adjusting microfluidic parameters; e.g., different fluid viscosities and flow rates (determined by Reynolds number and capillary number) can be used to control the particle size and number of nanoparticles [35].

Therefore, the aim of this study is to continuously prepare CPC nanosuspensions by microfluidic technology in a two-step method and systematically evaluate the physicochemical properties and oral bioavailability of curcumin–phospholipid complex nanosuspensions, including the intermediate (CPC) and curcumin nanoparticles (CUR-NPs), to provide a new idea and method for improving the bioavailability of curcumin and promoting its commercial production.

## 2. Materials and Methods

### 2.1. Materials

Curcumin (≥98%), polyvinylpyrrolidone k30 (PVP K30), sodium dodecyl sulfate (SDS), ethyl acetate (HPLC), and hexyl hydride were provided by Adamas-beta^®^, Shanghai, China. ß-Glucuronidase was provided by Sigma-Aldrich (Shanghai) Trading Co., Ltd., Shanghai, China. Soy lecithin was provided by AVT (Shanghai) Pharmaceutical Tech Co., Ltd., Shanghai, China. Methanol (HPLC), acetonitrile (HPLC), ethanol (AR), glacial acetic acid (AR), and other chemical reagents used were provided by Sinopharm Chemical Reagent Co., Ltd., Shanghai, China.

### 2.2. Animals

Pharmacokinetic studies in rats were conducted according to the guidelines of the Declaration of Helsinki and approved by the Animal Committee of Shanghai University of Traditional Chinese Medicine (Ethics: PZSHUTCM2411250002 and approved in June 2024). Male Sprague-Dawley (SD) rats weighing 200–240 g were provided by Shanghai Sipple-Bikai Laboratory Animal Co., Ltd., Shanghai, China, and were housed in the Shanghai University of Traditional Chinese Medicine Laboratory Animal Center (Shanghai, China). Prior to the start of the experiments, the animals were housed in environmentally controlled rearing chambers (temperature: 22 ± 2 °C, relative humidity: 45–60%) for 2–3 days with free access to standard laboratory feed and water. The animals were fasted 12 h prior to the experiment and had free access to water.

### 2.3. Methods

#### 2.3.1. CPC Preparation by Microfluidics

**Figure 1:**
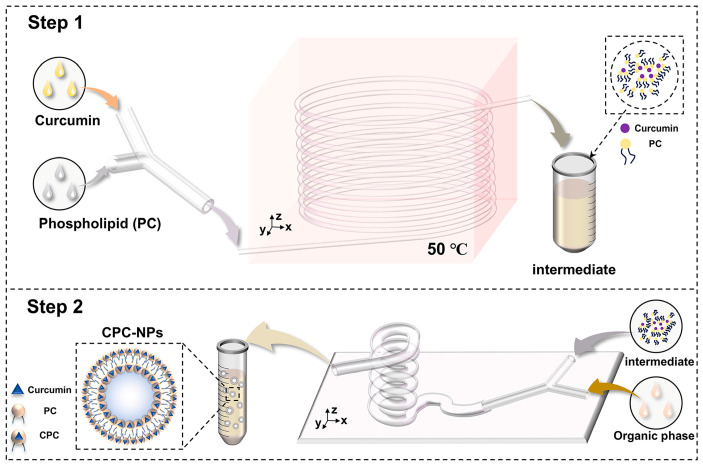
Schematic diagram of the preparation process of curcumin–phospholipid complex nanoparticles (CPC-NPs).

The preparation of CPC by microfluidics is shown in Figure 1. Curcumin and soy lecithin were dissolved in anhydrous ethanol and pumped into the microfluidic chip with a micro syringe pump at different flow rates, respectively. After heating for some time, the clarified solution obtained was evaporated, and the appropriate amount of n-hexane solution was added to make it re-dissolve, and the filtrate was filtered through a 0.45 um microporous filter membrane, and the filtrate was the n-hexane solution of CPC.

#### 2.3.2. CPC-NPs Preparation by Microfluidics

CPC-NPs were prepared on the basis of CPC. The organic (ethanol solutions containing CPC) and aqueous phases (deionized water containing PVP K30 and SDS) were injected into the microfluidic fittings at a certain flow rate using two channels of a microsyringe pump, respectively. The two phases play the role of shearing (aqueous phases) and sheared (ethanol solutions) in the microfluidic channel, mix thoroughly, and the nanosuspension of the curcumin–phospholipid complex flows out of the outlet of the device (Figure 1). In addition, CUR-NPs were prepared according to the same method and used to evaluate the pharmacokinetics [36].

#### 2.3.3. Dynamic Light Scattering (DLS) Characterization

CUR-NPs and CPC-NPs were measured using DLS with BeNano 90Zeta (Bettersize Instruments Ltd., Dandong, China). Three measurements of hydrodynamic diameter (Dh), polydispersity index (PDI), and ζ-potential were performed for each sample, and the results are expressed as mean and standard deviation (SD).

#### 2.3.4. Preparation Optimization

Before conducting the Box–Behnken design experiment (BBD), curcumin concentration, flow rate ratio, total flow rate, and stabilizer concentration on particle Dh and PDI were investigated through complete randomized design. On this basis, flow rate ratio (A), total flow rate (B), and stabilizer concentration (C) were selected as the independent variables, and particle Dh and PDI were selected as the dependent variables to design a BBD response surface experiment (three-factor and three-level) using Design-Expert 13^®^ software. Analysis of variance (ANOVA) was used to analyze the results, and the 3D surface diagram was drawn to predict the optimal parameters, which were verified by experiments. The three factors of the experimental design and their respective three levels are represented in Table 1.

#### 2.3.5. Infrared Spectral Characterization

Infrared spectral scanning of curcumin, phospholipid, a physical mixture of curcumin and phospholipid, and CPC using an FTIR spectrometer. Sample preparation by potassium bromide tableting: the sample is ground, mixed with potassium bromide, and then tableted. The spectral range was 400–4000 cm^−1^ with 32 scans and a resolution of 4 cm^−1^.

#### 2.3.6. Differential Scanning Calorimetry Characterization

Thermal analysis of samples using a 214-DSC calorimeter (NETZSCH, Selb, Germany) to determine. Curcumin, phospholipid, physical mixture of curcumin and phospholipid, CPC, CPC-NPs, and CUR-NPs were sealed in the aluminum crimp cell and heated at the speed of 10 °C/min from 30 °C to 200 °C in a nitrogen atmosphere (40 mL/min).

#### 2.3.7. X-Ray Powder Diffraction Characterization

XRD of curcumin, phospholipid, physical mixture of curcumin and phospholipid, CPC, CPC-NPs, and CUR-NPs were detected using a D8 ADVANCE X-ray diffractometer (Bruker, Billerica, MA, USA) and scanned at a diffraction angle range of 5–50° using a Cu-Ka radiation generator set at 40 mA and 40 kV, with a step length of 0.02° and a speed of 6°/min.

#### 2.3.8. Transmission Electron Microscopy

CPC-NPs and CUR-NPs were examined morphologically using TEM. A drop of 10 μL of CPC-NPs and CUR-NPs was applied to a carbon-coated grid (200 mesh, EMCN Co) that had been glow discharged for 60 s in air, and the grids were immediately negatively stained using uranyl acetate acid for 60 s. Grids were examined in an H-7800 operated at 80–120 kV.

#### 2.3.9. Drug Loading Capacity

CPC-NPs and CUR-NPs were diluted tenfold with an anhydrous ethanol solution, respectively, followed by centrifugation at 12,000 r/min for ten minutes, and the supernatant was injected and detected according to the HPLC analytical method. Details of the HPLC analytical methods are given in the Supplementary Materials.

DLC% = W1/W2 × 100%

where W1 and W2 are the weight of the drug in CPC-NPs (or CUR-NPs) and the weight of CPC-NPs (or CUR-NPs), respectively.

#### 2.3.10. Stability Study

The prepared CPC-NPs and CUR-NPs were divided into two aliquots; one was stored at room temperature, and the other was stored in a refrigerator at 4 °C; the Dh and PDI of the two samples were determined at 0, 4, 8, 24, 48, 72, and 96 h during the stability testing, respectively. In addition, the stability of CPC-NPs and CUR-NPs under physiological conditions was determined, i.e., CPC-NPs and CUR-NPs were dispersed in phosphate buffer solutions at pH 1.2 and pH 7.2, and their Dh and PDI were measured at 0, 2, 4, 6, and 12 h.

#### 2.3.11. Release Rate In Vitro

Release characterization of CPC, CPC-NPs, and CUR-NPs in release medium (phosphate buffer:ethanol = 7:3 at PH 1.2 to simulate gastric fluid environment and PH 6.8 to simulate intestinal environment) was carried out using the dialysis bag method, respectively [37,38,39,40,41]. One mL of CPC, CPC-NPs, and CUR-NPs dispersions were added to a ready-to-use dialysis bag with a cut-off relative molecular weight of 3500, respectively, and subjected to stirring in a 36 °C water bath with heat. One mL of release solution was taken at 15, 30, 45, 60, 90, 120, 240, 360, 480, 600, and 720 min after the start of the release, and the same volume and temperature of fresh release medium was added. The release solution was centrifuged at 12,000 r/min for 10 min, and the supernatant was taken for HPLC assay to calculate the cumulative drug release rate.

$$\mathrm{Cumulative}\;\mathrm{release}\;\mathrm{rate}\%=\sum_{0}^{t}\left(\frac{Ct}{C0}\right)\times 100$$

where *C*_0_ is the initial concentration of curcumin contained in the dialysis bag sample and *C_t_* is the concentration of curcumin in the phosphate buffer at each sampling time point.

#### 2.3.12. Hemolysis Assay

Hemolysis experiments were conducted to evaluate the bio-compatibility of CPC-NPs and CUR-NPs [40,42,43]. Saline and a 2% volume fraction of erythrocyte suspension were mixed as a negative control, and ultrapure water and 2% erythrocyte suspension were mixed as a positive control group. CUR, CPC, CUR-NPs, and CPC-NPs were dispersed in saline to a concentration of 1, 3, and 6 μg/mL, respectively. The above samples were mixed with 2% erythrocyte suspension (*v*/*v*, saline) from SD rats, incubated at 37 °C for 4 h, and then centrifuged at 1800 r/min for 10 min to observe whether hemolysis occurred. The supernatant was taken, and the absorbance value was measured at 570 nm [44], and the hemolysis rate was calculated (n = 3). The formula for calculating hemolysis rate: hemolysis rate = (A preparation − A negative − A blank)/(A positive − A negative).

#### 2.3.13. Organic Solvent Residues

The n-hexane solvent used in the preparation of CPC in this study is a class III solvent (solvents to be avoided) demonstrated in the ICH guidelines, and as a formulation for oral administration, the residual amount of n-hexane in the final product needs to be evaluated. Therefore, a gas chromatographic detection method was established to detect hexane residues in CPC and CPC-NPs using headspace injection.

#### 2.3.14. Pharmacokinetics Study

Twenty-four rats were randomly divided into four groups (N = 6) [45,46]. Curcumin (control), CPC, CPC-NPs, and CUR-NPs were administered orally at a dose of 200 mg/kg (based on the content of curcumin). Blood samples were collected at 15, 30, 45, 60, 120, 240, 360, 480, 600, 720, and 840 min after oral administration and stored in EDTA-treated anticoagulated centrifuge tubes. Plasma samples were centrifuged at 4 °C for 10 min at 3000 r/min, and the supernatant was stored at −80 °C in a refrigerator until analysis.

One hundred μL of 0.1 M ammonium acetate buffer containing 1000 Units of β-glucuronidase was added to a certain amount of drug-containing plasma [47,48]. The mixture was vortexed for 2 min to ensure thorough homogenization, followed by incubation at 37 °C in a water bath for 2 h to facilitate enzymatic hydrolysis of glucuronidated drug metabolites. Subsequently, the sample was extracted by adding 0.6 mL of ethyl acetate and vortexed for an additional 2 min. The mixture was centrifuged at 4 °C and 14,000 rpm for 10 min to separate the phases. The supernatant was then dried under vacuum at 50 °C. The residue was reconstituted with 100 μL of mobile phase and subjected to ultrasonication while vortexing for 2 min within ultrasonic for 1 min to ensure complete dissolution. Finally, this prepared solution was taken and analyzed using a validated HPLC assay.

HPLC analysis was performed on an Agilent 1200 HPLC system equipped with a C18 column (Pntulips BP-C18Plus, 5 μm × 4.6 mm × 250 mm column, GL Sciences Inc., Shanghai, China) and a UV detection wavelength of 426 nm. The mobile phase consisted of a mixture of acetonitrile and 4% glacial acetic acid (55:45, *v*/*v*) with a flow of 1.0 mL/min. The column temperature was 25 °C and the injection volume was 50 μL. The methodological validation was described in Supplementary Materials.

#### 2.3.15. Statistical Analysis

Measurement data obtained were expressed as the means with standard deviation and performed using SPSS^®^ (Version R26.0). The t-test was used to compare the means of two groups of measures, and analysis of variance (ANOVA) was used to compare the means of multiple groups of measures. When *p* > 0.05, it was considered that there was no statistically significant difference between the data; when *p* < 0.05, it was considered that there was a statistically significant difference between the data; when *p* < 0.01, it was considered that there was a statistically very significant difference between the data. The non-atrial model was analyzed using Phoenix^®^ software (Version 8.5.1) to obtain pharmacokinetic parameters.

## 3. Results

### 3.1. Preparation and Optimization Studies

Referring to previous studies [49], the principle that CPC is dissolved in hexane, but the free drug is not, was used to confirm the correct process of CPC preparation by microfluidic technology.

**Figure 2:**
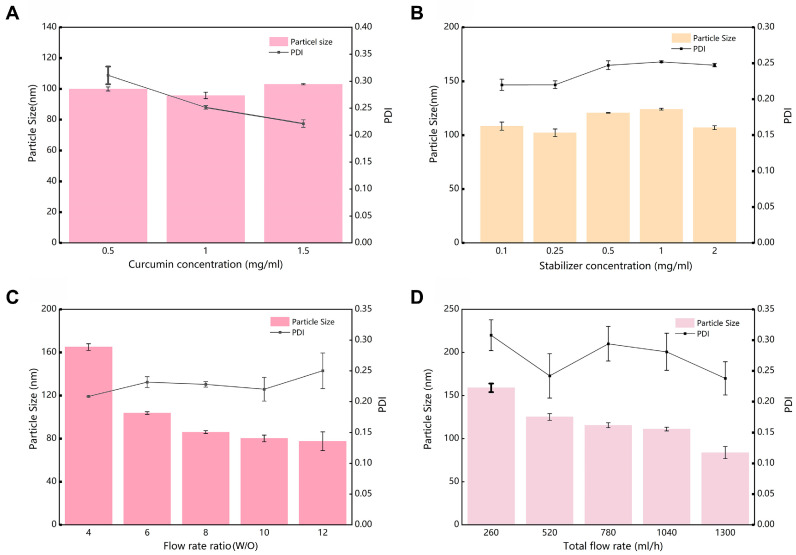
Summary of the results in the completely randomized design experiment. (**A**) Effect of curcumin concentration on nanoparticle particle Dh and PDI. (**B**) Effect of stabilizer (PVP K30) concentration on nanoparticle Dh and PDI. (**C**) Effect of flow rate ratio (W/O) on nanoparticle particle Dh and PDI. (**D**) Effect of total flow rate on nanoparticle particle Dh and PDI.

The optimization results of the completely randomized design of CPC- NPs, including curcumin concentration, stabilizer concentration, flow rate ratio, and total flow rate, were shown in Figure 2A–D. Dh of CPC-NPs was independent over the range of curcumin concentrations tested from 0.5 to 1.5 mg/mL, so the concentration of curcumin was determined to be 1.5 mg/mL (Figure 2A). Under different stabilizer concentrations, CPC-NPs could maintain narrowly dispersed (PDI < 0.3), and with the increase of stabilizer concentration, the particle Dh of CPC-NPs changed from decreasing to increasing to decreasing (Figure 2B). Meanwhile, with the increase of flow rate ratio (W/O) and total flow rate, the particle Dh of CPC-NPs decreased, and both maintained a good mono-dispersity coefficient (Figure 2C,D). The above results indicated that the flow rate ratio as well as the total flow rate are the key process parameters for the preparation of CPC-NPs by microfluidics, which can be varied to adjust the particle Dh.

Subsequently, the 3-factor 3-level BBD response surface study (17 tests in total) was designed using Design-Expert 13^®^ software with flow rate ratio (A), total flow rate (B), and stabilizer concentration (C) as independent variables. Due to variations in factor combinations, the particle Dh ranged from 74 nm to 136 nm (Table 2). The F-value of the preferred model was 47.18 (*p* < 0.0001), which met the acceptability criteria of the model, and the non-significance of the misfit term (0.7542) confirmed the acceptability of the model. The 3.48% coefficient of variation value and good consistency between the R-squared values and the adjusted R-squared values (Table 3), both indicating that the model was reliable and sophisticated.

**Figure 3:**
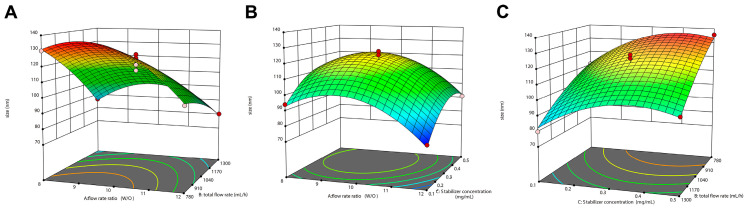
Three-dimensional surface for two-factor interactive effect. (**A**) Interaction of flow rate ratio (W/O) and total flow rate. (**B**) Interaction of flow rate ratio (W/O) and stabilizer concentration. (**C**) Interaction of total flow rate and stabilizer concentration.

**Figure 4:**
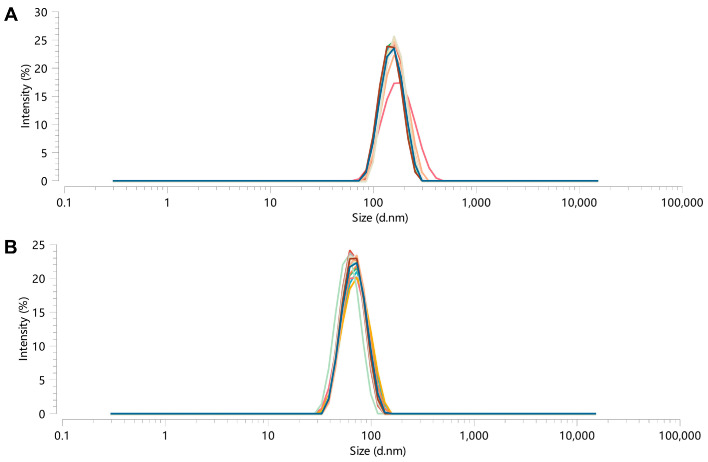
Particle Dh distribution of CPC-NPs (**A**) and CUR-NPs (**B**) (N = 3).

Then, the validation test was carried out (Table 4), and the 3D surface for the two-factor interactive effect was plotted (Figure 3). The results showed that the actual measured values were basically consistent with the predicted values (Figure 4). And the particle Dh of the prepared CUR-NPs was (180 ± 9.88) nm with (−22.2 ± 0.7) mV in the zeta potential.

### 3.2. Stability Study

**Figure 5:**
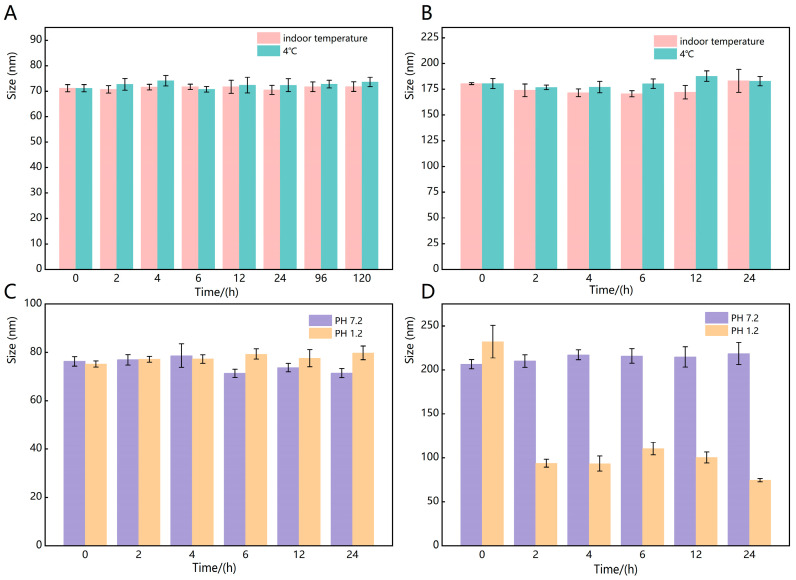
Stability of CPC-NPs (**A**) and CUR-NPs (**B**) stored at room temperature and 4 °C; CPC-NPs (**C**) and CUR-NPs (**D**) stored at PH 1.2 and 7.2.

**Figure 6:**
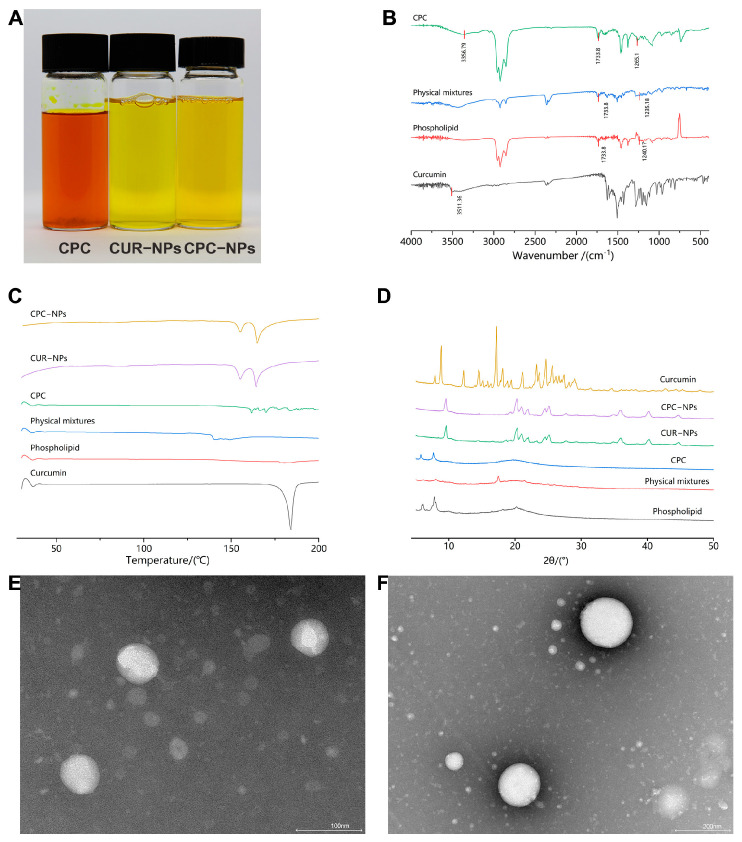
Visualization and characterization of different preparations. (**A**) Visual images of CPC, CUR-NPs, and CPC-NPs. (**B**) FT-IR spectrum of curcumin, phospholipid, physical mixture of curcumin and phospholipid, and CPC. (**C**) DSC thermograms of curcumin, phospholipid, physical mixture of curcumin and phospholipid, CPC, CUR-NPs, and CPC-NPs. (**D**) XRD patterns of curcumin, phospholipid, physical mixture of curcumin and phospholipid, CPC, CUR-NPs, and CPC-NPs. (**E**) TEM results for CPC-NPs. (**F**) TEM results for CUR-NPs.

No obvious aggregation and sedimentation of the samples were found, and the results of particle Dh measurements at different time points showed good stability over five days for CPC-NPs and acceptable stability over three days for CUR-NPs, both at room temperature and at 4 °C (Figure 5A,B). And the PDI of CPC-NPs or CUR-NPs in both room temperature and 4 °C environments was <0.3 during the stability tests. The results of Figure 5C show that CPC-NPs can maintain good stability in both PH 1.2 and PH 7.2 environments; the results of Figure 5D show that CUR-NPs can maintain relatively good stability in PH 7.2 environments, and the particle size changes are larger in PH 1.2 environments, which may indicate that strong acidic environments can destroy the structure of CUR-NPs. The solution appearance of the three preparations, CPC, CUR-NPs, and CPC-NPs, is shown in Figure 6A.

### 3.3. Drug Loading Capacity

The results of drug loading are shown in Table 5. The results of drug loading showed that the drug loading capacity of CPC-NPs was about 2.5 times that of CUR-NPs, indicating that CPC-NPs had a more obvious superiority over CUR-NPs.

### 3.4. Hexane Residue

The linear equations of the n-hexane assay were 0.265~12.716 μg/mL (r > 0.999), and the detection limit of n-hexane was 0.1060 μg/mL calculated according to the signal-to-noise ratio (SN ≥ 3). The results of n-hexane determination in CPC and CPC-NP samples according to this assay showed that n-hexane residues in CPC and CPC-NPs were less than 290 ppm (ICH Guidelines for n-hexane residues in CPCs and CPC-NPs). More detailed information can be found in the Supplementary Materials.

### 3.5. Infrared Spectral (IR) Characterization

The FT-IR spectroscopy showed that the hydroxyl absorption peak of the CPC based on microfluidic control technology was 3357 cm^−1^, and the phenolic hydroxyl absorption peak located at 3511 cm^−1^ had a lower wavelength shift compared with the curcumin monomer. Meanwhile, the C=O and P=O absorption peaks were 1733 cm^−1^ and 1265 cm^−1^, respectively, and the corresponding C=O and P=O absorption peaks were 1734 cm^−1^ and 1240 cm^−1^, respectively. These peaks shifted to higher wave numbers compared with the corresponding absorption peaks of soybean lecithin (Figure 6B). Compared with the pre-complexed state, the phenolic hydroxyl group was red-shifted, and P=O absorption peaks were blue-shifted, indicating that the -OH in curcumin is connected to P=O in phospholipids through hydrogen bonding and the CPC successfully complexes.

### 3.6. Differential Scanning Calorimetry (DSC) Characterization

The DSC thermal analysis plots showed that the free drug had a single melting point at around 184 °C, indicating the crystalline nature of curcumin, while CPC did not show any significant melting peaks, indicating that the drug changed from crystalline to amorphous form after complex formation. On the other hand, the characteristic peak of free drug produced by pure endothermy at 184 °C in CPC-NPs and CUR-NPs disappeared, and two distinct endothermy peaks were shown at 155 °C and 164 °C, indicating the formation of both nanoparticles (Figure 6C).

### 3.7. X-Ray Powder Diffraction (XRD) Characterization

The XRD analysis plots showed that the diffraction pattern of free drug was strongly crystalline with more sharp peaks indicating a crystalline form, whereas in CPC these characteristic peaks disappeared and were replaced by one blunt and broad amorphous peak (Figure 6D). This may be due to the complexation of curcumin with phospholipids as well as changes in structural morphology. Meanwhile, the diffraction patterns of both CPC-NPs and CUR-NPs indicated that the crystal type of curcumin was also changed, and both were likely to be a “mixture” of crystalline and amorphous forms. Physically mixed samples provided an objective control, showing that the characteristic summit of curcumin is largely preserved when curcumin is not complexed with phospholipids.

### 3.8. Transmission Electron Microscopy (TEM) Characterization

The TEM results showed that the particle size of CPC-NPs was 57–65 nm and that of CUR-NPs was 152–187 nm. AND the TEM results of CPC-NPs showed that CPC-NPs had a relatively rounded spherical-like structure with uniform distribution, clear edges, and no adhesion aggregation. CUR-NPs were basically the same as CPC-NPs (Figure 6E,F).

### 3.9. Hemolysis Assay

**Figure 7:**
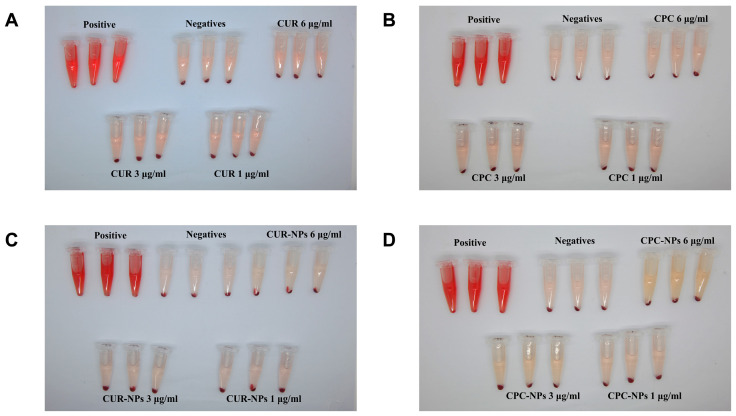
Hemolysis effect of CUR (**A**) and CPC (**B**); CUR-NPs (**C**); and CPC-NPs (**D**).

The results showed that the hemolysis rates of CUR, CPC, CPC-NPs, and CUR-NPs were less than 5%. Interestingly, the hemolysis rate of CUR-NPs turned out to be less than 0, which may imply that CUR-NPs can inhibit the rupture of erythrocytes and maintain the stability of erythrocyte membranes to a certain extent, thus reducing the degree of hemolysis, and this protective effect showed a dose dependence within 1–6 μg/mL, and the CPC-NPs (1–3 μg/mL) presented a similar protective effect to CUR-NPs. However, CPC-NPs showed an opposite trend at 6 μg/mL versus 3 μg/mL; this may be due to the presence of SDS (hemolytic agent) in the prescription, as the concentration of Cur increased, so did the concentration of SDS, increasing the rate of hemolysis. Comparing the results of CUR and CUR-NPs, CPC, and CPC-NPs, we found that the preparation of prodrugs or intermediates in the form of nano-formulations can increase their biocompatibility to some extent. Overall, these data suggest to some extent that microfluidics-based preparation of CPC, CPC-NPs, and CUR-NPs is quite biocompatible. The detailed results of the hemolysis experiments are shown in Table 6 and Figure 7 and Figure S5.

### 3.10. In Vitro Release

**Figure 8:**
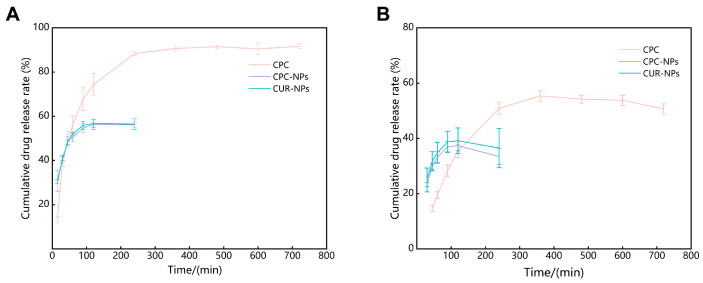
(**A**) Release profiles of CPC, CPC-NPs, and CUR-NPs in simulated gastric fluid (PH = 1.2). (**B**) Release profiles of CPC, CPC-NPs, and CUR-NPs in a simulated intestinal environment (pH = 6.8).

The in vitro release profiles of CPC, CPC-NPs, and CUR-NPs are shown in Figure 8 and Table 7. The results showed that, in the environment of simulated gastric fluid (pH 1.2), CPC was rapidly released within 4 h after administration and reached release equilibrium after 6 h; the cumulative release rate of CPC was 91.65 ± 1.07%; CPC-NPs were rapidly released within 90 min of administration and essentially reached equilibrium at 2 h, and the cumulative release of CPC-NPs was 56.15 ± 1.22%. Like CPC-NPs, CUR-NPs had similar release characteristics and eventually reached release equilibrium; the cumulative release was 56.73 ± 1.79%. In the simulated intestinal fluid environment (pH 6.8), the release characteristics of each group were similar to those of the simulated gastric fluid environment, although the cumulative release was decreased. The reason for not reaching 100% cumulative release of CPC, CPC-NPs, and CUR-NPs may be due to the decomposition of curcumin in the in vitro release medium as suggested in previous studies [13,50]. The cumulative release of curcumin in simulated intestinal fluid (pH 6.8) was lower than that in simulated gastric fluid (pH 1.2), which may be due to the faster breakdown of curcumin in neutral and alkaline environments [51]. The cumulative release rates of CPC, CPC-NPs, and CUR-NPs were fitted to the zero-level, one-level, Higuchi, and Korsmeyer–Peppas equations, respectively, and the correlation coefficients, R, were calculated, with the closer the value of R to 1, the higher the agreement with the release equations. The in vitro releases of CPC, CPC-NPs, and CUR-NPs in the gastric fluid environment (pH 1.2) and in the intestinal environment (pH 6.8) were all first-order release equations, indicating that drug release is mainly dependent on diffusion.

### 3.11. Pharmacokinetics

**Figure 9:**
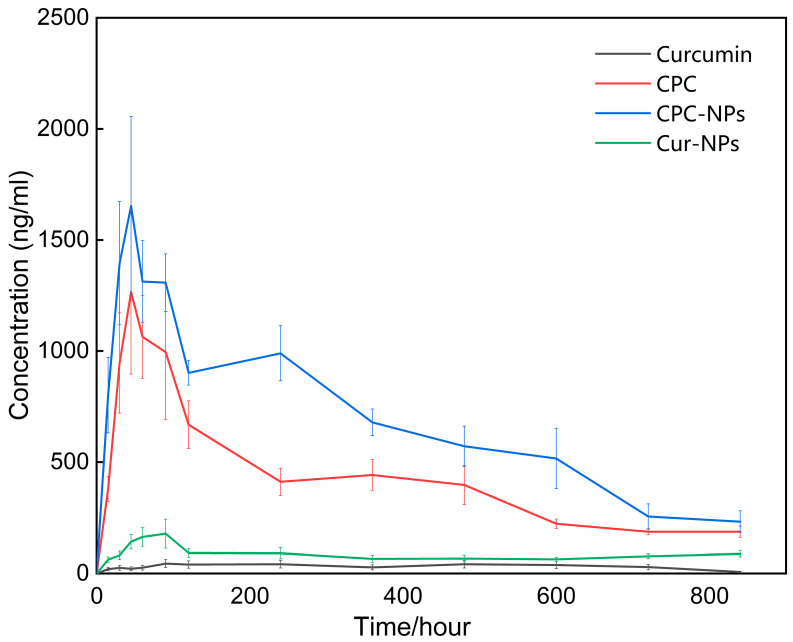
Drug-time curves of curcumin-free drug, CPC, CUR-NPs, and CPC-NPs.

Non-atrial model analysis was performed using Phoenix^®^ software (Version 8.5.1) to obtain pharmacokinetic parameters and drug-time curves as shown in Table 8 and Figure 9. The results showed that the C_max_ of curcumin, CUR-NPs, CPC, and CPC-NPs were 133.60 ± 28.10, 270.23 ± 125.42, 1894.43 ± 672.65, and 2163.87 ± 777.36 ng/mL; the AUC_0–t_ of curcumin, CUR-NPs, CPC, and CPC-NPs were 936.99 ± 201.83, 1155.46 ± 340.38, 5888.79 ± 1073.32, and 9494.28 ± 1863.64 ng/mL/h, respectively. Compared with the curcumin group, the CPC group had an approximately 14-fold increase in C_max_ and a 6-fold increase in AUC; the CPC-NPs group had an approximately 16-fold increase in C_max_ and a 10-fold increase in AUC. On the other hand, the pharmacokinetic parameters of CUR-NPs showed only negligible improvement over free drug, probably due to the larger particle size of CUR-NPs compared to CPC-NPs and the different loading strategy. These results indicated that CPC and CPC-NPs formulations could enhance the oral bioavailability of curcumin.

Meanwhile, given the advantages of both the phospholipid matrix and nanoparticles in promoting drug absorption, the drug peak time of CPC-NPs (0.79 ± 0.37 h) was much earlier than that of the free drug (6.17 ± 4.09 h) and even better than that of CPC (1.00 ± 0.25 h) with the advantage of the phospholipid matrix only. The drug half-life of CPC-NPs was basically consistent with that of CPC. What needs illustration is that due to the low bioavailability of free drug and CUR-NPs, the objective individual differences will lead to some outliers in the original data, which do not meet the test provisions of normal distribution. After the exclusion of outliers, the sample size is less than the minimum value that can be used for statistics, and it is difficult to conduct analysis.

## 4. Discussion

In this research, we proposed for the first time a two-step preparation strategy of CPC-NPs using a microfluidic system in order to overcome the obstacles of curcumin, such as poor water solubility, poor absorption, and low bioavailability [9,10]. Previously, a pure CPC was also considered for development, but its poor oral stability and semisolid nature undermined its research and development value [30]. Pharmacokinetics studies confirmed this hypothesis, showing that the bioavailability of CPC-NPs was about 10-fold higher than that of free drug, while CPC alone only exerted half of the effects of the above nano-formulations. To further support the necessity for this preparation strategy, CUR-NPs that did not need to be prefabricated into CPC were compared synchronously and found to be much less capable of improving bioavailability than CPC or even basically comparable to the free drug.

From the point of view of the drug release mechanism, there are several possible differences in the effects of these formulations on the bioavailability of curcumin. The better performance of CPC than that of the free drug may be related to the phospholipid in CPC, which can promote the absorption and solubility of curcumin [52]. Additionally, due to the sharp increase in the specific surface area of the nano-form [53], the contact area between CPC-NPs and the absorption site of the gastrointestinal tract is expanded, and the absorption ability of curcumin is improved. Meanwhile, the outer shell composed of polyvinylpyrrolidone K30 and sodium dodecyl sulfate enhances the intragastric stability of the formulation [54] and collaborates with internal phospholipids to complete the delivery of curcumin. These results not only support the application value of multi-layer drug-loaded nano-formulations [55] but also provide new ideas for the application of continuous formulation characteristics of microfluidic systems.

In the process of microfluidic platform design, the inner diameters of the vast majority of the reference devices are at the micron level [56,57,58,59], which considerably limits the drug preparation ability and increases the blockage risk [60]. In this study, the flow channels with an inner diameter slightly larger than 1 mm purchased from the market were used to assemble the microfluidic device used in the experiments. By adjusting the stabilizer concentration, the two-phase flow rate ratio, and the total flow rate, particles with particle Dh ranging from 70 nm to 200 nm could be prepared with good mono-dispersity and reproducibility. These results also indicate that micron-level inner diameters are not a prerequisite for the preparation of high-quality nano-formulations. From an economical and commercial point of view, it is possible to avoid the high cost of accessories by using connection lines and adapters that can be mass-produced.

To further match with the actual commercial environment and R&D conditions, more convenient analytical methods were also emphasized. After curcumin is absorbed into the blood, the REDOX metabolism (phase I) and the binding metabolism (phase II) are carried out in sequence so that the binding reaction of glucose acidification or sulfation occurs under the action of enzymes, and a considerable part of the free curcumin is converted into the bound state in the blood [61]. Therefore, based on the principle of reverse enzymolysis, the decomposition of bound curcumin in blood samples by β-glucuronidase makes it possible to use HPLC alone to complete the analysis work. In this study, a systematic HPLC assay that requires pre-enzymolysis of blood samples was developed and validated, providing a low-cost assay option. Although this strategy can only measure the total curcumin content in blood (the amount of free curcumin and curcumin–glucosinolate conjugates), it still has the potential to be generalized from a pharmacological point of view. Recent studies have shown that glucosinolate conjugates of curcumin exhibit anti-tumor activities [62,63] and can play an important role in the NF-κB-mediated therapeutic effect of curcumin in vivo [64]. Studies have shown [65] that curcumin has a sufficiently safe dose range and that the cytotoxicity of curcumin–phospholipid nanoparticles originates from the release of curcumin and is not induced by phospholipids. As for PVP K30, which is commonly used as an excipient in oral formulations, there is no significant toxicity, so we believe that CPC, CPC-NPs, and CUR-NPs mentioned in this study may have a sufficiently safe dose range. Studies have shown that the metabolites of Cur are distributed in different tissues, mainly in the spleen, liver, lungs, and kidneys [66]. Detailed information related to toxicity and in vivo distribution will be disclosed in a subsequent pharmacodynamic study.

However, this study still has several limitations. First, a comprehensive pharmacodynamic evaluation is not mentioned in this article, and in view of the need to carry out patent applications in China, these works will be disclosed in the subsequent expression of results. Secondly, considering the superior bioavailability properties, it is necessary to investigate the drug release mechanism of CPC-NPs. Furthermore, the feasibility of commercial-scale production of this strategy should be further verified.

## 5. Conclusions

This study is the first to propose and systematically characterize a two-step strategy for the preparation of CPC-NPs using a microfluidic system, which was compared with free drug, CPC, and CUR-NPs. Despite its promise, microfluidics in the formulation field still faces bottlenecks in large-scale production (e.g., chip clogging, equipment stability) and a lack of standardization. This study shows that high-precision microfluidic chips are no longer a prerequisite for research and that these slightly larger structures have more potential for industrial applications. Importantly, pharmacokinetic studies confirmed that the bioavailability of CPC-NPs was superior to that of free drug, CPC, and CUR-NPs, suggesting the potential of this formulation strategy for practical production. Pharmacodynamic evaluation of anti-inflammatory, anti-oxidation, and neuroprotection needs to be confirmed in follow-up studies.

## Figures and Tables

**Table 1:** Factors and levels in the BBD for CPC-NP preparation.

Factor	Name	Low Level	Medium Level	High Medium
A	flow rate ratio	8	10	12
B	total flow rate (mL/h)	780	1040	1300
C	Stabilizer concentration (mg/mL) ^1^	0.1	0.3	0.5

^1^ Stabilizer concentration refers to the concentration of PVP K30.

**Table 2:** Summary of the results of the 17 tests in the BBD trial.

Std	Run	Factor A	Factor B	Factor C	Dh/nm	PDI
1	14	8	780	0.3	130	0.26
2	1	12	780	0.3	100	0.25
3	12	8	1300	0.3	86	0.21
4	2	12	1300	0.3	80	0.22
5	15	8	1040	0.1	94	0.23
6	10	12	1040	0.1	74	0.20
7	6	8	1040	0.5	107	0.26
8	4	12	1040	0.5	91	0.27
9	17	10	780	0.1	111	0.29
10	3	10	1300	0.1	80	0.23
11	7	10	780	0.5	136	0.27
12	8	10	1300	0.5	96	0.27
13	16	10	1040	0.3	119	0.26
14	5	10	1040	0.3	125	0.31
15	9	10	1040	0.3	123	0.29
16	11	10	1040	0.3	115	0.27
17	13	10	1040	0.3	119	0.29

**Table 3:** Summary of regression analysis of BBD-fitted models.

Source	Sequential *p*-Value	Lack of Fit *p* Value	R^2^	Adjusted R^2^	Predicted R^2^	C.V. %
Quadratic	<0.0001	0.7452	0.9838	0.9629	0.9179	3.48

**Table 4:** Optimization of predicted and actual values of CPC-NPs.

Value	Factor A	Factor B	Factor C	Dh/nm	PDI	Zeta Potential/mV	C.V. %
Predicted value	11.63	1238.91	0.12	74	/	/	1.90
Validating value	11.60	1238.00	0.12	72 ± 1.37	0.23 ± 0.05	38.2 ± 0.9	

**Table 5:** Drug loading results for CPC-NPs and CUR-NPs (mean ± SD, N = 3).

Formulation	Curcumin Content-μg/mL	DLC%
CPC-NPs	121.21 ± 0.56	10.61 ± 0.24
CUR-NPs	142.61 ± 1.35	4.33 ± 0.10

**Table 6:** Results of hemolysis experiments with curcumin, CUR-NPs, CPC, and CPC-NPs (mean ± SD, N = 3).

Formulations	Concentration-μg/mL	Hemolysis/%
CUR	1	0.40 ± 0.16
	3	0.50 ± 0.19
	6	0.91 ± 0.10
CPC	1	1.30 ± 0.05
	3	1.63 ± 0.17
	6	1.85 ± 0.07
CPC-NPs	1	−0.23 ± 0.13
	3	−0.67 ± 0.12
	6	1.67 ± 0.13
CUR-NPs	1	−1.62 ± 0.69
	3	−2.34 ± 0.23
	6	−3.11 ± 0.09

**Table 7:** Release kinetic parameters (r2) for CPC, CPC-NPs, and CUR-NPs data obtained using several mathematical models.

Simulated Environment	Formulations	Release Models	R^2^
Stomach	CPC	Zero order	0.531
		**First order**	**0.99** **2**
		Higuchi	0.756
		Korsmeyer–Peppas	0.831
	CPC-NPs	Zero order	0.466
		**First order**	**0.981**
		Higuchi	0.607
		Korsmeyer–Peppas	0.727
	CUR-NPs	Zero order	0.390
		**First order**	**0.982**
		Higuchi	0.698
		Korsmeyer–Peppas	0.814
Intestine	CPC	Zero order	0.717
		**First order**	**0.98** **1**
		Higuchi	0.839
		Korsmeyer–Peppas	0.858
	CPC-NPs	Zero order	0.391
		**First order**	**0.961**
		Higuchi	0.596
		Korsmeyer–Peppas	0.681
	CUR-NPs	Zero order	0.237
		**First order**	**0.955**
		Higuchi	0.489
		Korsmeyer–Peppas	0.578

**Table 8:** Pharmacokinetic parameters of curcumin, CUR-NPs, CPC, and CPC-NPs (mean ± SD, N = 6).

Formulations	T_max_/h	T_1/2_/h	C_max_/(ng/mL)	AUC_0–t_/(ng/mL × h)
Curcumin	6.17 ± 4.09	NA ^1^	133.60 ± 28.10	936.99 ± 201.83
CUR-NPs	NA ^1^	NA ^1^	270.23 ± 125.42	1155.46 ± 340.38
CPC	1.00 ± 0.25	5.85 ± 1.50	1894.43 ± 672.65	5888.79 ± 1073.32
CPC-NPs	0.79 ± 0.37	5.18 ± 2.69	2163.87 ± 777.36	9494.28 ± 1863.64

^1^ The original data had outliers, which did not conform to the normal distribution, and the sample size was ≤3 after removing the outliers.

## Data Availability

The datasets used and analyzed during the current study are available from the corresponding author on reasonable request.

## Supplementary Materials

The following supporting information can be downloaded at: https://www.mdpi.com/article/10.3390/pharmaceutics17030395/s1, Figure S1: Results of the Exclusive Examination; Figure S2: Standard curve; Figure S3: n-hexane standard curve; Figure S4: Chromatograms of CPC and CPC-NPs; Figure S5: Results of hemolysis test; Figure S6: Representative HPLC chromatograms of Curcumin and the IS (Nimodipine). (a) Blank plasma. (b) Blank plasma spiked with LLOQ (10 ng/mL) concentration of Curcumin and IS (Nimodipine). (c) Plasma sample collected at oral Curcumin in rats.; Figure S7: HPLC standard curve of curcumin in rat plasma; Table S1: Results of Precision Examination (n = 6); Table S2: Results of Accuracy Examination (n = 6); Table S3: Results of Stability Examination; Table S4: Standard curve; Table S5: Inter-batch- or intra-batch precision and accuracy for curcumin from quality control samples (Mean ± SD, n = 5); Table S6: Recovery and matrix effects for the analytes from quality control samples (Mean ± SD, n = 5); Table S7: Dilution integrity in rat plasma (Mean ± SD, n = 5); Table S8: Stability at room temperature for 24 h (Mean ± SD, n = 5).

## Abbreviations

The following abbreviations are used in this manuscript:

CPC	Curcumin–Phospholipid Complex
CPC-NPs	Curcumin–phospholipid Complex Nanosuspensions
CUR-NPs	Curcumin Nanosuspensions
HPLC	High-Performance Liquid Chromatography
IR	Infrared Spectroscopy
DSC	Differential Scanning Calorimetry
XRD	X-ray Diffraction
TEM	Transmission Electron Microscope
PDI	Poly Dispersity Index
Zeta	Zeta potential
IS	Internal Standard
