# Supplementary material for: Physicochemical Characterization and Oral Bioavailability of Curcumin–Phospholipid Complex Nanosuspensions Prepared Based on Microfluidic System
Source: Pharmaceutics. 2025 Mar 20;17(3):395. doi: 10.3390/pharmaceutics17030395 (PMC11946702; doi:10.3390/pharmaceutics17030395)
Supplement: Supplementary file 1 [file pharmaceutics-17-00395-s001.zip › pharmaceutics-3534530-supplementary.pdf]

Supplementary Materials

Validation of in vitro analytical methods for curcumin

1. Method validation

The method validation was mainly based on the Chinese Pharmacopoeia 9012 Guidelines for Validation of Methods for Quantitative Analysis of Biological Samples (2020 edition). The method was fully validated selectivity, standard curve, accuracy, precision and stability.

1.1 Curcumin standard solution preparation

Take appropriate amount of curcumin standard, weigh it precisely, transfer it to 100 ml brown volumetric flask, dissolve it in anhydrous ethanol and determine the volume, shake well to obtain the ethanol solution of curcumin standard with the concentration of 228.732 μg/ml (mother solution).Measure 1 ml of curcumin mother liquor into 5 ml brown volumetric flask, dilute with anhydrous ethanol to the scale, shake well, that is, the concentration of 45.746 μg/ml curcumin control solution. The same method as above, the concentration of 22.873 μg/ml, 11.437 μg/ml and 2.287 μg/ml of curcumin control solution were obtained by sequential dilution respectively.

1.2 Chromatographic conditions

HPLC analysis was performed on a Agilent 1200 HPLC system equipped with a C18 column (Pntulips BP-C18Plus, 5 μm × 4.6 mm × 250 mm column, GL Sciences Inc., Chinese) and a UV detection wavelength of 426 nm. The mobile phase consisted of a mixture of acetonitrile and 2% glacial acetic acid (65:35, v/v) with a flow of 1.0 ml/min, The column temperature is 35°C and the injection volume is 10μl.

1.3 Selectivity

10 μl of curcumin standard solution, curcumin phospholipid complex test solution and blank solvent were taken respectively, and the samples were injected and detected according to the above chromatographic conditions.

1.4 Linearity

Curcumin standard solutions with concentrations of 228.732 μg/ml, 45.746 μg/ml,

22.873 μg/ml, 11.437 μg/ml and 2.287 μg/ml were injected into the samples and detected in accordance with the above assay conditions in this section, and the linear regression equations were fitted with the concentration as the abscissa X and peak area as the abscissa Y. The results were shown in the following table.

1.5 Precision

Take the appropriate concentration of curcumin standard solution, according to the above conditions in this section of the detection of continuous injection 6 times, according to the results of the calculation of RSD value

1.6 Accuracy（Sample recovery rate）

Appropriate amount of the known content of curcumin phospholipid complex test material was added to the low, medium and high concentrations of curcumin standard solution, and the recovery was calculated.

1.7 Stability

Take the appropriate concentration of curcumin phospholipid complex test solution, respectively, at 0, 2 h, 4 h, 6 h, 12 h, 24 h, 48 h, 72 h after preparation according to the above detection conditions into the sample detection, according to the results of the calculation of RSD value.

2.Result

2.1 Selectivity

The results of curcumin standard solution, curcumin phospholipid complex test solution and blank solvent were shown in Fig S1. The results indicated that soy lecithin did not interfere with curcumin peak detection and the method was of good specificity.

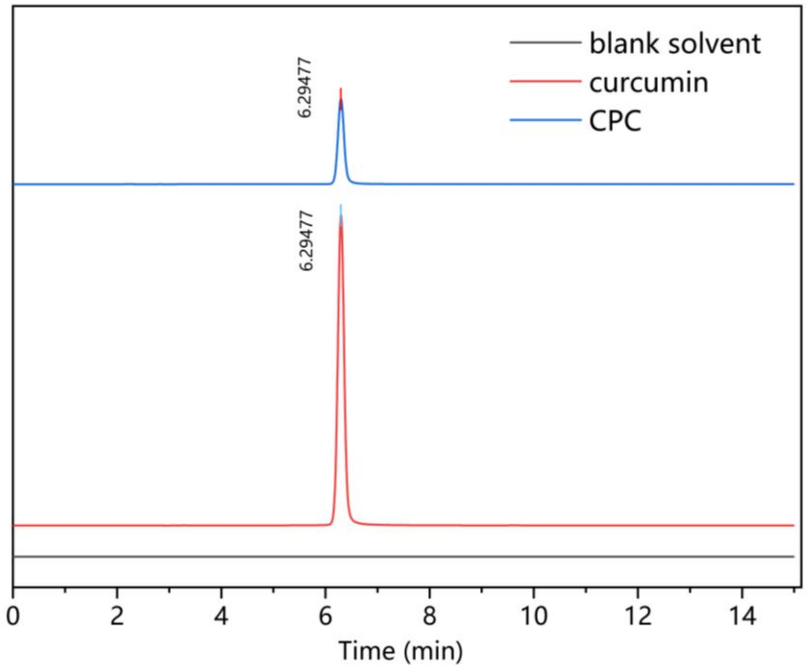

Figure S1. Results of the Exclusive Examination

2.2 Linearity

Linear fitting was carried out with different concentrations as horizontal coordinates X and corresponding peak areas as vertical coordinates Y. The results were as follows

The results showed a good linear relationship in the concentration range of 2.287 μg/ml-228.73 μg/ml with the linear regression equation: y=85.648x+198.89 and the regression coefficient r=0.9996.

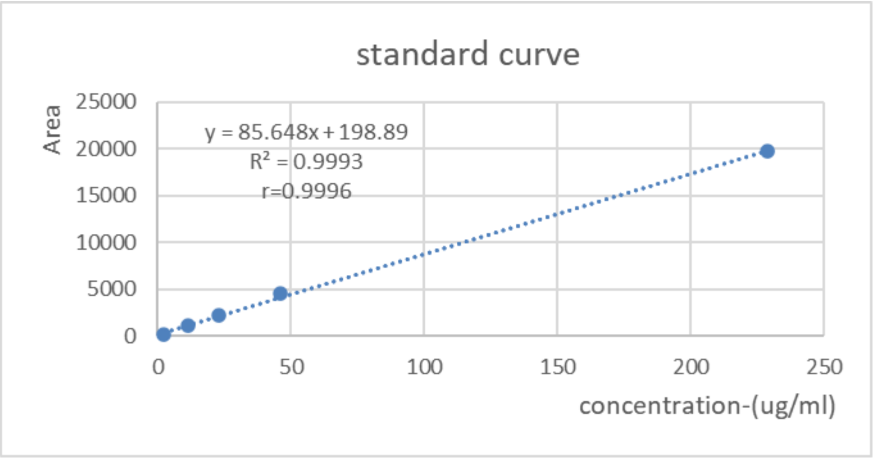

Figure S2. Standard curve

2.3 Precision

The precision results of curcumin HPLC analyses are shown in Table S1., indicating

good instrumental precision.

Table S1. Results of Precision Examination (n=6)

	**1**	**2**	**3**	**4**	**5**	**6**	**RSD%**
Area	2430.0	2432.9	2437.1	2439.6	2441.0	2442.9	0.18

2.4 Accuracy (Sample recovery rate)

The Accuracy results of curcumin HPLC analyses are shown in Table S2., indicating the accuracy of this method is good.

Table S2. Results of Accuracy Examination (n=6)

**concentration of CPC(ug/ml)**	**Concentration added (ug/ml)**	**Calculated concentration (ug/ml)**	**Actual concentration (ug/ml)**	**Accuracy%**
14.23	50	64.23	66.45	103.46
14.23	100	114.23	117.19	102.59
14.23	120	134.23	136.78	101.90

2.5 Stability

The results of the stability study of the curcumin phospholipid complex are shown in Table S3., suggesting that the stability of the sample within 72 h is as required.

Table S3. Results of Stability Examination

**Time /h**	**0**	**2**	**4**	**6**	**8**	**12**	**24**	**48**	**72**	**RSD %**
Area	2433.3	2477.0	2460.8	2510.9	2469.9	2472.5	2432.6	2461.9	2427.0	1.00

Validation of analytical methods for n-hexane residues

1. Materials and Instruments

1.1 Materials

Deionized Water, Guangzhou Watsons Food & Beverage Co., Ltd., Guangzhou, China), N, N-dimethylformamide (DMF), ANPEL Laboratory Technologies (Shanghai) Inc., hexane(⩾99.5%), Aladdin Holdings Group Co., Ltd

1.2 Instruments

Gas chromatograph: 6890N-7697A (FID detector/headspace injector), Agilent Technologies Inc.;

Ultrasonic Cleaner: SB-500DTY, NingBo Scientz Biotechnology Co.,Ltd；

Electronic balance: AB104-N, Mettler-Toledo International Trading (Shanghai) Co., Ltd.；

Electronic balance: XS205DU, Mettler-Toledo International Trading (Shanghai) Co., Ltd.；

2. Method

2.1 Chromatographic conditions

column： Agilent-DB-624, (30 mm*0.25 mm*1.4 μm).Programmed temperature increase: start at 40°C, hold for 10 min, then increase to 100°C at 6°C/min, hold for 3 min, then increase to 200°C at 20°C/min, hold for 2 min. Headspace injection, headspace vial equilibrium temperature 80°C, equilibration time 30 min, quantitation loop temperature 90°C, transfer line temperature 100°C, GC cycle time 40 min, injection time 1 min. Hydrogen flame ionisation detector, detector temperature 250°C, inlet temperature 200°C, carrier gas nitrogen at a flow rate of 1.0 ml/min, split flow, split ratio 10:1.

2.2 Linear solution preparation

Weigh 21.3mg of n-hexane control, weigh it precisely, put it into a 20 ml measuring flask (1-2 ml of DMF was added in advance in the flask), and make a solution of 1000 μg per 1 ml with N,N-dimethylformamide to obtain the mother solution of the control.Take an appropriate amount of the mother liquor of the control product, and use 20% N,N-dimethylformamide solution to formulate a series of concentration.Measure 5.0 ml of each concentration of n-hexane control solution into a 20 ml headspace bottle and seal it, that is to say, the control solution was obtained.

2.3 Sample preparation

Take about 0.3 g of CPC and CPC-NPs respectively, weigh them precisely, put them in a 20 ml headspace bottle, add 5 ml of 20% N,N-dimethylformamide solution, seal it, and ultrasonicate (power 300 W, frequency 40 kHz) for 10 min, then it was obtained.

3. Linearity

The headspace gas of each linear solution was measured precisely at 1 ml each, injected into the gas chromatograph, and the peak area was recorded.The peak area of the substance to be measured was taken as the vertical coordinate, and the concentration (C) of each substance was taken as the horizontal coordinate for linear fitting, and a linear equation was obtained (R2>0.99), the standard curve is shown in Table S4., and the linear graph is shown in Fig.S3. The results showed that n-hexane had a good linearity in the range of 0.265~12.716 μg/mL.

Table S4. Standard curve.

concentration-μg/mL	0.265	1.060	2.119	5.298	12.716
Peak area	2.811	7.949	11.454	26.329	63.910
linear equation	y=4.8665x+1.6053	R^2	0.9988		

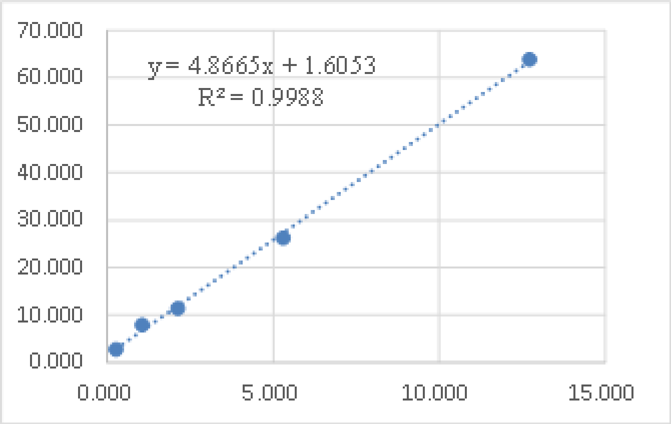

Figure S3. n-hexane standard curve

3.1 Lower limit of detection

The mother liquor of the control product was diluted step by step and measured according to the gas chromatographic conditions, the ratio of signal intensity to noise intensity was recorded, and the concentration at which the ratio of signal intensity to noise intensity was ≥3 was taken as the limit of detection (LOD), and the experimental results showed that the LOD concentration of n-hexane was

0.1060 μg/ml.

3.2 Determination of n-hexane residues

CPC and CPC-NPs were determined according to the determined assay method, and the results showed that the n-hexane concentration in both CPC and CPC-NPs was below the detection limit, and the related chromatograms are shown in Figure S4.

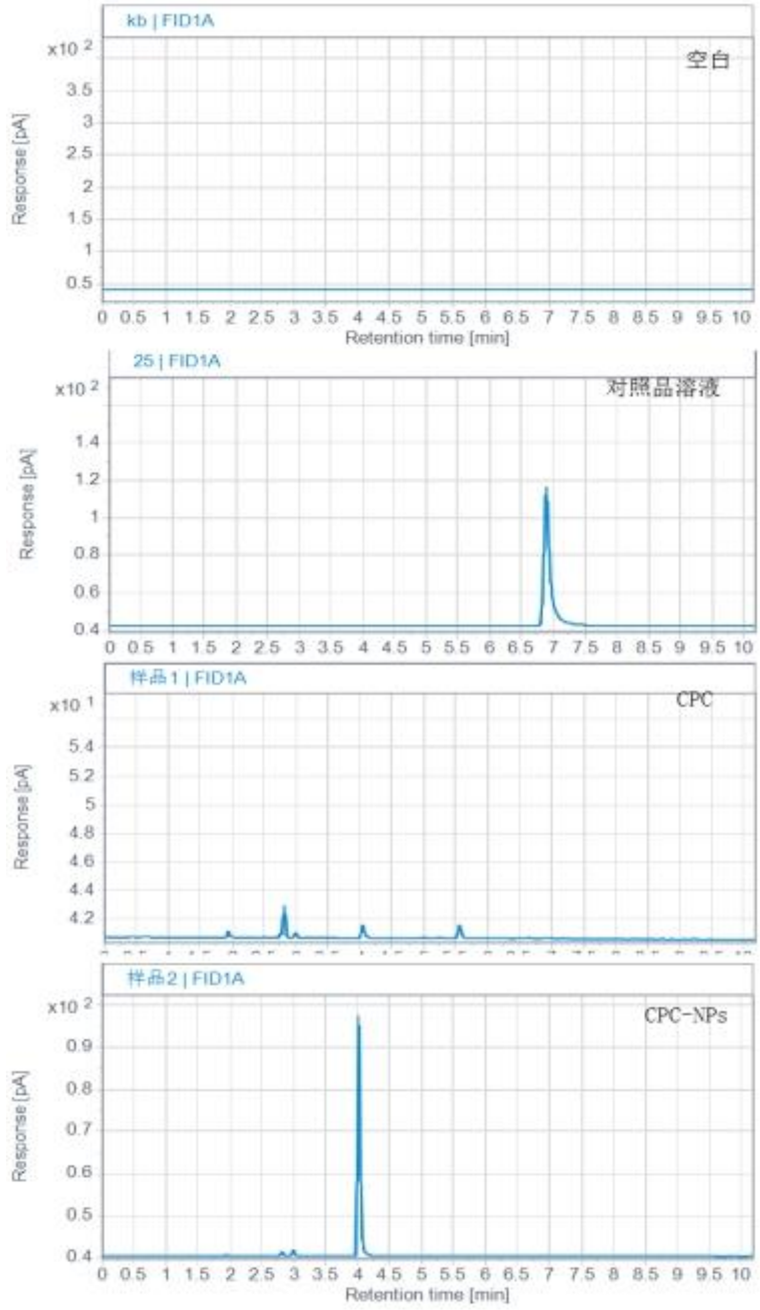

Figure S4. Chromatograms of CPC and CPC-NPs.

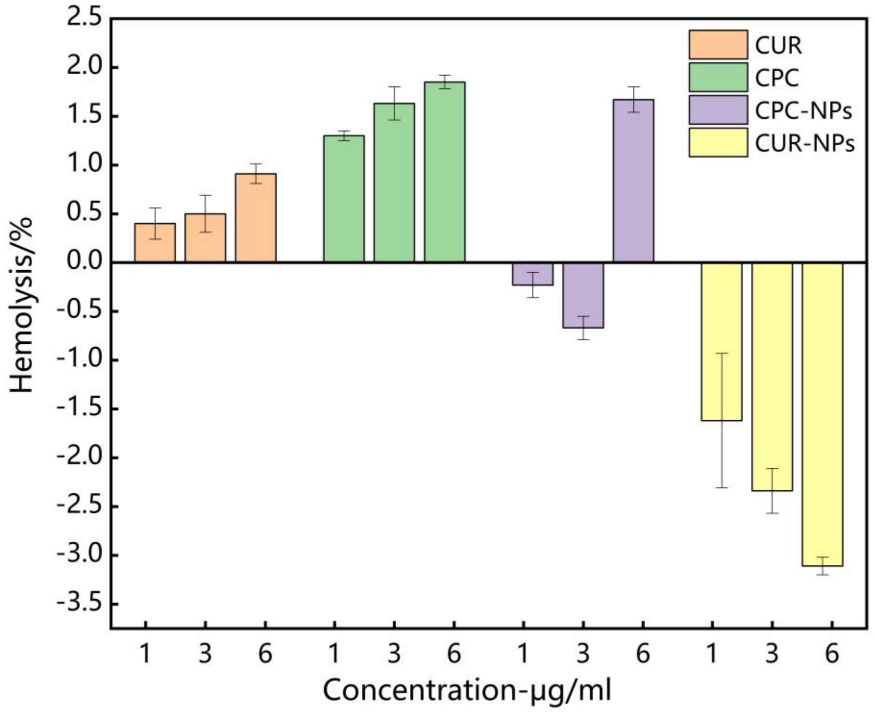

Figure S5. Results of hemolysis test.

Validation of in vivo analytical methods for curcumin

3. Method validation

The method validation was mainly based on the Chinese Pharmacopoeia 9012 Guidelines for Validation of Methods for Quantitative Analysis of Biological Samples (2020 edition). The method was fully validated in blank rat plasma, including selectivity, residue, lower limit of quantification, standard curve, accuracy, precision, extraction recovery, matrix effect, and stability.

3.1 Preparation of calibration standards and quality control samples

Curcumin control solution (original stock solution) was prepared in methanol at a concentration of 4 μg/mL. The original stock solution was diluted with methanol to

obtain a series of standard working solutions of curcumin control. The internal standard Nimodipine was dissolved in methanol to obtain a working solution containing 0.6 mg/mL Nimodipine internal standard. All solutions were stored at 4 °C and brought back to room temperature before use. Corrected samples with final concentrations of 1000 ng/mL, 700 ng/mL, 300 ng/mL, 100 ng/mL, 50 ng/mL, and 10 ng/mL were prepared by adding different concentrations of curcumin methanol control working solution and internal standard nimodipine working solution to rat blank plasma. Quality control samples (QC) at concentrations of 30, 500 and 800 ng/mL were prepared in a similar manner.

3.2 Selectivity, carry-over, and lower limit of quantification

Selectivity is assessed by comparing the chromatograms of six different rat blank plasma samples. If an interfering fraction is present, the response value of the interfering fraction should be less than 20% of the lower limit of quantification for curcumin and less than 5% of the response for the internal standard. Carry-over of curcumin and nimodipine are calculated by injecting a blank sample after analysis of a high-concentration sample or a calibration standard sample, with Carry-over in the blank sample following the high-concentration sample not exceeding 20% of the lower limit of quantification and not exceeding 5% of the internal standard. The lower limit of quantification (LLOQ) is calculated according to the signal-to-noise ratio (S/N) and should satisfy $S/N > 10$.

3.3 Linearity

Linearity was assessed by measuring the calibration curve in plasma in three separate runs, using the curcumin concentration as x and the ratio of the response value of curcumin to the response value of the nimodipine (internal standard) as y. The calibration curves were performed in three separate runs. The back-calculated concentration of the calibration specimen should be within ±15% of the nominal value, and the back-calculated concentration of the lower limit of quantification should be within ±20% of the nominal value, and at least 75% of the calibration specimens should

meet the above criteria.

3.4 Accuracy and precision

Inter-batch (three batches) and intra-batch precision and accuracy were assessed in five replicates at four concentration levels (10, 30, 500 and 800 ng/mL). Freshly prepared calibration and quality control samples were used for all precision and accuracy studies. Precision was evaluated by relative standard deviation (RSD) and accuracy was expressed as relative error RE (measured value/stated value*100%). The RSD should be less than 15% (20% for LLOQ) and the RE should be within ±15% (20% for LLOQ) at each concentration level.

3.5 Extraction recovery and matrix effect

Extraction recoveries were calculated at four concentration levels (LLOQ, low, medium, and high concentrations, i.e., 10,30, 500, and 800 ng/mL) by comparing the analytical results of the extracted samples with those of the corresponding blank extracts spiked with the internal standard and analytes after extraction, and the reproducibility of extraction recoveries was expressed in terms of RSD, which should be less than15%.

The matrix effect was expressed by calculating the internal standard normalization of matrix factors at four concentration levels (LLOQ, low, medium and high concentrations, i.e., 10, 30, 500 and 800 ng/mL). The internal standard normalization of matrix factors were calculated by calculating the ratio of the peak area in the presence of the matrix (measured from a blank matrix extraction followed by the addition of the analyte and internal standard) to the ratio of the corresponding peak area in the absence of matrix (a pure solution of the analyte and the internal standard) and the RSD of internal standard normalization of matrix factors should be less than 15%.

3.6 Stability

The corresponding calibration curve that was obtained based on freshly prepared

calibration samples was employed to obtain the measured concentrations. The short-term stability of prepared samples was evaluated using the QC samples. The QC samples were placed in an auto-sampler that was set at room temperature for 24 h. The analytes were considered stable when the concentration biases were below 15% for different levels.

3.7 Dilution reliability

Oral administration of the drug resulted in a drug-containing rat plasma sample that was diluted 3-fold and 10-fold with blank rat plasma, respectively, and the analyte required a concentration above the limit of quantification (LLOQ). The corresponding calibration curve obtained using a freshly prepared calibration sample was used to obtain the measured concentration, and the dilution reliability was tested using a quality control sample, with at least 5 determinations for each dilution factor. The accuracy and precision of the dilution should be less than 15%.

4. Result

4.1 Selectivity, carry-over, and lower limit of quantification

As shown in Figure S6., curcumin and nimodipine showed good separation in the biological matrix species with clear peak morphology and the endogenous substances in the blank plasma did not interfere with the determination of either. The LLOQ of curcumin was set at 10 ng/mL. In rat plasma, the intra-batch accuracy and precision of LLOQ was respectively 100.50% and 1.83%, while the inter-batch accuracy and precision was 95.79% and 8.78%, respectively. The detailed results were shown in Table 1.The lower limit of quantification (LLOQ) is calculated according to the signal-to-noise ratio (S/N) and satisfies S/N > 10. In addition, curcumin and nimodipine were not detected when a blank sample was injected after the injection of a high-concentration QC sample, suggesting that curcumin and nimodipine residues were in compliance with the requirement.

(a)

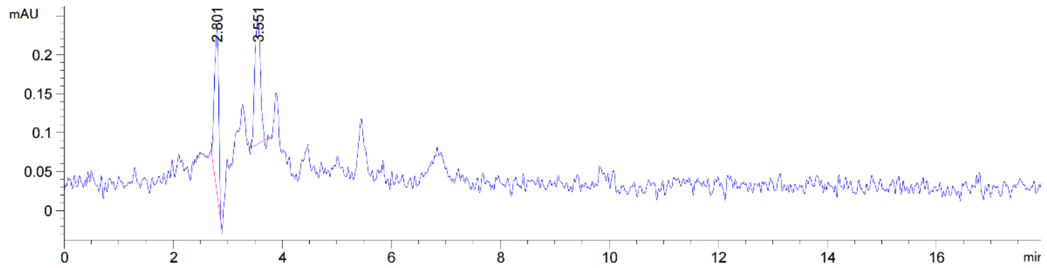

(b)

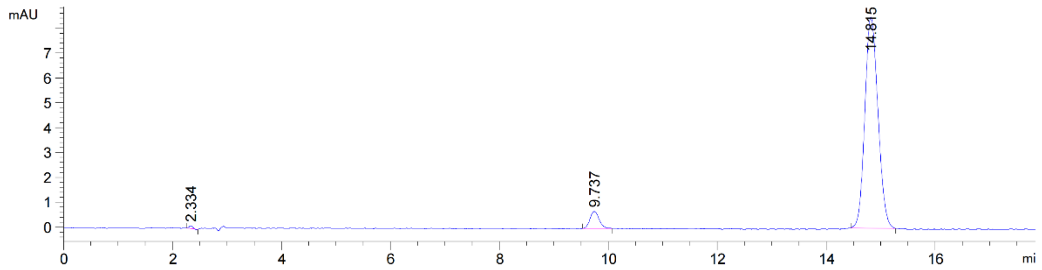

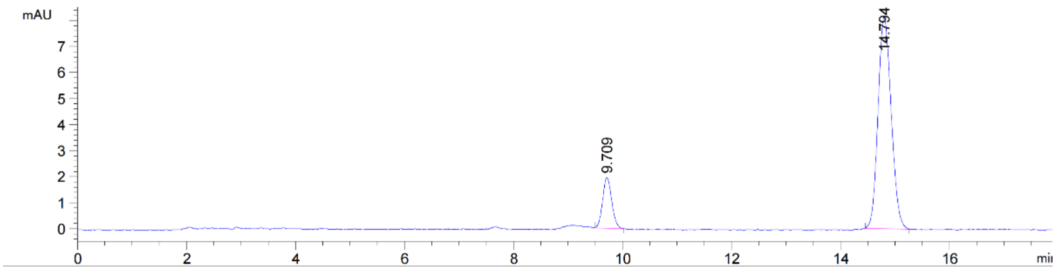

Figure S6. Representative HPLC chromatograms of Curcumin and the IS (Nimodipine). (**a**) Blank plasma. (**b**) Blank plasma spiked with LLOQ (10 ng/mL) concentration of Curcumin and IS (Nimodipine). (**c**) Plasma sample collected at oral Curcumin in rats.

4.2 Linearity

Linearity was assessed by measuring the calibration curve in plasma, using the curcumin concentration as x and the ratio of the response value of curcumin to the response value of the nimodipine (internal standard) as y. As shown in Figure S7., curcumin showed good linearity in rat plasma with a linear range of 10-1000 ng/mL. The calibration curves were performed in three separate runs. The back-calculated concentration of the calibration specimen should be within ±15% of the nominal value, and the back-calculated concentration of the lower limit of quantification should be within ±20% of the nominal value, and at least 75% of the calibration

specimens should meet the above criteria.

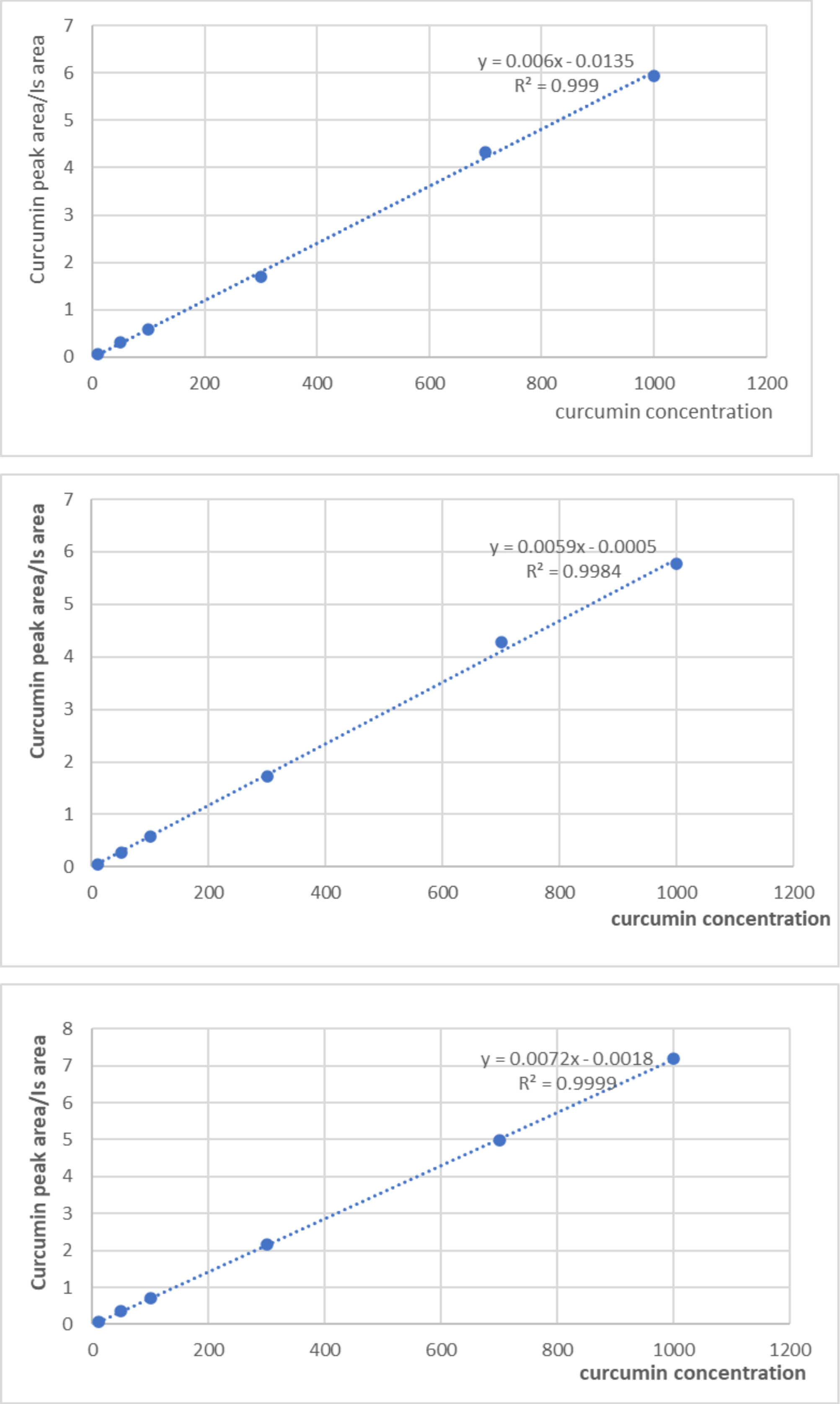

Figure S7. HPLC standard curve of curcumin in rat plasma

4.3 Precision and accuracy

The intra-batch precision range of curcumin in the rat plasma was 1.83%-5.29 % and the inter-batch precision range was 3.85%-8.78%, while the intra-batch accuracy range was 91.30%~100.50% and the inter-batch accuracy range was 95.15%-97.61%. The detailed results were shown in Table S4.

Table S5. Inter-batch- or intra-batch precision and accuracy for curcumin from quality control samples (Mean±SD, n = 5)

**Inter-batch- or intra-batch**	**Nominal concentrations(ng/mL)**	**Measured concentrations(ng/mL)**	**Precision (%)**	**Accuracy (%)**
intra-	10	10.05±0.18	1.83	100.50
batch	30	30.02±1.59	5.29	100.07
	500	456.49±9.44	2.07	91.30
	800	798.17 ±22.86	2.86	99.77
inter-	10	9.58±0.84	8.78	95.79
batch	30	28.54±1.34	4.70	95.15
	500	488.06±23.63	4.84	97.61
	800	761.26±29.31	3.85	95.16

4.4 Extraction recovery and matrix effect

The average recovery of curcumin and nimodipine was respectively 96.18%-101.68% in rat plasma and the RSD was 1.11% and 5.81% in rat plasma. The results show that this method has a high and stable extraction recovery. The detailed results were shown in Table 2.

The matrix effects on curcumin and nimodipine was 95.75%-102.84% in rat plasma and the RSD was 1.63% and 5.41% in rat plasma. The results suggested that ion

suppression or enhancement was not significant with the HPLC method. The detailed results were shown in Table S5.

Table S6. Recovery and matrix effects for the analytes from quality control samples (Mean±SD, n = 5)

Nominal concentrations (ng/mL)	Recovery(%)	RSD(%)	Matrix effects(%)	RSD(%)
10	101.68±4.13	4.07	95.75±5.18	5.41
30	98.99±4.15	4.20	102.84±6.81	6.62
500	100.07±1.11	1.11	95.78±2.37	2.48
800	96.18±5.59	5.81	100.12±1.63	1.63

4.5 dilution reliability

Quality control samples of different concentrations were prepared and diluted 3-fold and 10-fold, respectively, so that the concentrations after dilution were 10 ng/mL and 500 ng/mL, respectively. The analytical accuracy of the diluted samples was 90.96% to 102.79%, and the precision was 2.23% and 3.44%, respectively. The results show that diluting the plasma sample 3-fold and 10-fold does not affect the accurate determination of the sample concentration. The detailed results of the dilution integrity were shown in Table S6.

Table S7. Dilution integrity in rat plasma (Mean±SD, n = 5)

Dilution factor	Nominal concentration (ng/mL)	Measured concentration (ng/mL)	Calculated concentration (ng/mL)	Precision (%)	Accuracy (%)
3	30	10.36±0.23	31.09	2.23	103.65
	1500	458.52±10.44	1375.56	2.28	91.70
10	100	10.28±0.26	102.79	2.51	102.79
	5000	454.81±15.66	4548.07	3.44	90.96

4.6 Stability

Stability is expressed by the ratio of the concentration of the sample after 24 hours of storage at room temperature to the concentration of the sample at the time of the initial detection of the sample. As shown in Table S7., the stability of QC samples (at four concentrations, i.e., 10,30, 500, and 800 ng/mL) was 94.79%-96.48%. The results show that it was stable at room temperature for up to 24 hours after the samples were prepared.

Table S8. Stability at room temperature for 24 hours (Mean±SD, n = 5)

**Storage time/h**	**Nominal concentrations (ng/mL)**	**Measured Concentrations (ng/mL)**	**Precision (%)**	**Accuracy (%)**	**Stability(%)**
0	10	10.06±0.30	2.95	100.56	/
	30	31.47±0.41	1.30	104.90	
	500	499.84±10.90	2.18	99.97	
	800	786.01±8.21	1.04	98.25	
24	10	9.53±0.27	2.85	95.31	94.79
	30	30.36±0.55	1.83	101.21	96.48
	500	478.34±11.05	2.31	95.67	95.70
	800	754.75±13.11	1.74	94.34	96.02

Answers to several questions about the ARRIVE Guide 2.0

5.1 Study design

The experimental unit was each rat.

5.2 Sample size

The number of animals in each group was determined to be 5 or 6 regarding similar pharmacokinetic studies[1,2] in the literature and in the present study there were three preparations and curcumin prodrugs, hence a total of four groups of six experimental animals each were divided into.

5.3 Inclusion and exclusion criteria

5.4 Randomisation

In this study, random allocation in Excel software was used to assign all experimental animals to 4 groups, the method of randomizing the sequence i.e.: using excel software, firstly, Mr. 24 numbers in order of size, such as A1-A24 are 1, 2, 3, 4, 5, 6...,24 respectively, followed by the use of the function = SORTBY(A3:A13, RANDARRAY (COUNTA (A3: A13)), the A column in the order of the 24 numerical values randomly disrupt the order of allocation into four groups.

To minimize potential confounding factors to the greatest extent possible, the experiment was performed by the same experimenter for all blood sampling operations, and all animal cages are placed in the same area and kept under the same conditions to ensure that all experimental groups were affected by the source of the variant in approximately the same way.

5.5 Blinding

For each animal, 2 different researchers were involved, the first researcher was responsible for the gavage during the experiment as well as for taking blood, which could not be grouped in a hidden way due to the large differences in the shapes of the preparations, and the second researcher was responsible for the fact that, when the plasma samples were collected, compiled in serial numbers, and subjected to a simple pre-processing procedure, the person did not know which preparation had been administered orally to that experimental animal treatment, and the grouping.

5.6 Outcome measures

The following metrics were evaluated in this study: C_{max} (peak blood concentration), AUC (area under the curve). The primary outcome metric in this study was AUC , and the secondary outcome metric was C_{max}.

5.7 Statistical methods

For Cmax and AUC datas, SPSS software was first used to determine if there were any outliers, and if so, they were excluded. If the sample size after exclusion was $n \geqslant 5$, the data analysis could continue, if the sample size after exclusion was $n < 4$, the group was reported as missing in the manuscript and did not participate in the subsequent established analyses.

5.8 Experimental procedures

Bonded gloves, silicone gloves, disposable syringes (purchased from Contrail brand), sterile surgical cotton balls, and 1.5 ml EDTA anticoagulation tubes were required before the start of the experiment. The experimental animals were fasted for about 12 hours before starting the experiment but were allowed to drink freely. The experiments were performed in a suitable laboratory as specified. The jugular vein blood sampling method was used for this experiment because it is less harmful to rats. At the beginning of the experiment, 0h blood samples were first taken with a disposable syringe, followed by oral administration of the drug, after which samples were taken at intervals of 15, 30, 45, 60, 120, 240, 360, 480, 600, 720, 840 min.

6.1 Animal care and monitoring

As pharmacokinetic experiments were to be performed and the use of anaesthetics would have an impact on drug metabolism, analgesia without anaesthetics was chosen during the experiments.Although it is impossible to use anaesthetics to reduce the pain of rats in the experiments due to the nature of the study, the researchers will try their best to alleviate the pain of the animals in other ways, such as: if there is any blood coming out of the jugular vein after blood sampling, press a cotton ball to stop the bleeding in a timely manner; when grabbing the rats to keep them in place, care should be taken to avoid using excessive force, and do not pinch their neck to avoid asphyxiation and death.

No adverse events

Humane endpoints: rapid loss of 15-20% of original body weight; marked depression and unresponsiveness to loud repelling.

References:

1. Adiwidjaja, J.; Sasongko, L. Effect of Nigella sativa oil on pharmacokinetics and pharmacodynamics of gliclazide in rats. *Biopharm. Drug. Dispos.* **2021**, *42*, 359-371, doi:10.1002/bdd.2300.
2. Shahid, M.; Ahmad, A.; Raish, M.; Bin Jardan, Y.A.; Alkharfy, K.M.; Ahad, A.; Abul Kalam, M.; Ahmad Ansari, M.; Iqbal, M.; Ali, N.; et al. Herb-drug interaction: Effect of sinapic acid on the pharmacokinetics of dasatinib in rats. *Saudi. Pharm. J.* **2023**, *31*, 101819, doi:10.1016/j.jsps.2023.101819.
